# Notes and News

**Published:** 1900-05-19

**Authors:** 


### Notes and News.

HOSPITAL MEETINGS.

Homes for Working Boys in London.—Meeting at nine o'clock p.m., on Monday, May 21st, at Queen's Hall, Langham Place.

Paddington Green Children's Hospital.—Annual Meeting at five o'clock p.m., on Thursday, May 24th.

North-Eastern Hospital for Children.—Annual Meeting at half-past four o'clock p.m., on Thursday, May 24th.

East London Hospital for Children.—Festival Dinner at half-past seven o'clock p.m., on Friday, May 25th, at Victoria Hall, Hotel Cecil.

Medical Society of London.—Conversazione at half-past eight o'clock p.m., on Monday, May 21st, at 11, Chandos Street, Cavendish Square.

Newsvendors' Benevolent and Provident Institution.—Festival Dinner on May 22nd, at the Whitehall Rooms.

Many cases of small-pox have appeared in Canada lately.

Dr. Leonard Hill and Dr. Patrick Manson have been recommended by the Council of the Royal Society for election as Fellows.

It is reported that a Jewish rag merchant has been seized with the plague at Smyrna. In consequence, persons arriving there have to undergo an examination.

Dr. P. L. Benson, M.A., M.D., R.U.I., D.P.H.Camb., has been reappointed medical officer of health for the rural district of Buckingham.

H.R.H. the Princess of Wales has graciously consented to open the National Bazaar on May 24th. This function will take place at the Empress Rooms, Royal Palace Hotel, and adjoining grounds, which extend over some five acres. H.R.H. Field-Marshal the Duke of Cambridge, K.G., will open the Bazaar on May 25th. General Sir George White will open the Bazaar on May 26th.

A deputation from the bicycle organisations have approached Mr. Ritchie with a view to securing greater railway facilities for bicyclists at less cost than at present. The grievance is a real one. It is surprising that English cyclists do not do most of their touring in France, where bicycles are free on the railways, and receive reasonable care, where luggage is conveniently expedited without the owners, the majority of roads are admirable, and wayside refreshments are excellent and cheap.

Sir George White will address the 340 residents of the Homes for Working Boys at the thirtieth anniversary of the society at Queen's Hall, Langham Place, on May 21st. Other speakers will be the Dean of Norwich and the Rev. Marcus Rainsford. Office of the homes, 12, Buckingham Street, Strand.

The reformation in the administration of Addenbrooke's Hospital, Cambridge, is proceeding. The managers are now seeking a suitable candidate to fill the post of superintendent. This is a very important step, as the post is only instituted so far as an experiment. We learn that a local clergyman offered his services at a nominal remuneration. It is not likely, however, that the Advisory Council will elect anyone who has had no experience within a hospital.

A new infirmary has just been opened in connection with the workhouse at Basingstoke. It consists of a large block three storeys high, the administration being as usual in the centre, with wings for male and female patients on either side. On each floor is a ward containing 16 beds and two small separate wards, with bathroom and the usual offices. On the south side of the wards are balconies and verandahs, with iron ladder fire escapes. Good accommodation is provided for the nurses in the administration block. The cost of the buildings was little over £10,000.

According to a German medical officer attached to an ambulance with the Boer forces some of the Boers shot themselves with their own rifles in order to be released from military service, and he states as an interesting psychological fact that these "accidents" always happened a day before a battle was expected. He adds that some soldiers wounded in the extremities continued to fight, and were even able to ride to the dressing tent without support, but that the majority of the Boers when slightly wounded took the opportunity of withdrawing from the battle-field.

There seems a chance that the difficulties in the way of legislating for midwives may be still further enhanced by the General Medical Council turning sulky and declining the functions proposed to be handed over to it. The Council, having been asked its opinion, suggested certain amendments which have not been adopted in their entirety by the Grand Committee, and while some people, who know their Parliament, regard the getting of the money to pay the Council as the great difficulty, others are ready to suggest that the Council is "huffed," and will decline the work even for a consideration. We doubt it.

We learn that several changes have recently taken place in the medical staff of University College. Dr. Sydney Ringer has retired from the Holme Professorship of Clinical Medicine, and has been succeeded by Dr. F. T. Roberts. Mr. Christopher Heath has resigned the Holme Professorship of Clinical Surgery, and is succeeded by Mr. Rickman J. Godlee. Dr. Ringer and Mr. Heath have been appointed consulting physician and consulting surgeon respectively to University College Hospital. Dr. Roberts, on taking up the Holme Professorship, has resigned the Professorship of the Principles and Practice of Medicine, and the Council have selected Dr. G. Vivian Poore to succeed him. Lastly, Dr. H. Batty Shaw has been appointed an assistant physician to University College Hospital.

